# Thyroid Hormone Levels of Pregnant Inuit Women and Their Infants Exposed to Environmental Contaminants

**DOI:** 10.1289/ehp.0800219

**Published:** 2009-01-29

**Authors:** Renée Dallaire, Gina Muckle, Éric Dewailly, Sandra W. Jacobson, Joseph L. Jacobson, Torkjel M. Sandanger, Courtney D. Sandau, Pierre Ayotte

**Affiliations:** 1 Public Health Research Unit, Centre Hospitalier Universitaire de Québec-CHUL, Québec City, Québec, Canada; 2 Department of Social and Preventive Medicine, Laval University, Québec City, Québec, Canada; 3 School of Psychology, Laval University, Québec City, Québec, Canada; 4 Department of Psychiatry and Behavioral Neurosciences, Wayne State University School of Medicine, Detroit, Michigan, USA; 5 Department of Obstetrics and Gynecology, and Psychology, Wayne State University School of Medicine, Detroit, Michigan, USA; 6 Norwegian Institute for Air Research, The Polar Environmental Centre, Tromsø, Norway; 7 Trium Environmental Solutions, Inc., Cochrane, Alberta, Canada

**Keywords:** cohort, cord blood, Inuit, metabolites, organochlorines, pentachlorophenol, polychlorinated biphenyls, pregnant women, prenatal exposure, thyroid hormones

## Abstract

**Background:**

An increasing number of studies have shown that several ubiquitous environmental contaminants possess thyroid hormone–disrupting capacities. Prenatal exposure to some of them, such as polychlorinated biphenyls (PCBs), has also been associated with adverse neurodevelopmental effects in infants.

**Objectives:**

In this study we examined the relationship between exposure to potential thyroid hormone–disrupting toxicants and thyroid hormone status in pregnant Inuit women from Nunavik and their infants within the first year of life.

**Methods:**

We measured thyroid hormone parameters [thyroid stimulating hormone (TSH), free thyroxine (fT_4_), total triiodothyronine (T_3_), thyroxine-binding globulin (TBG)] and concentrations of several contaminants [PCB-153, hydroxylated metabolites of PCBs (HO-PCBs), pentachlorophenol (PCP) and hexachlorobenzene (HCB)] in maternal plasma at delivery (*n* = 120), in umbilical cord plasma (*n* = 95), and in infant plasma at 7 months postpartum (*n* = 130).

**Results:**

In pregnant women, we found a positive association between HO-PCBs and T_3_ concentrations (β = 0.57, *p* = 0.02). In umbilical cord blood, PCB-153 concentrations were negatively associated with TBG levels (β = −0.26, *p* = 0.01). In a subsample analysis, a negative relationship was also found between maternal PCP levels and cord fT_4_ concentrations in neonates (β = −0.59, *p* = 0.02). No association was observed between contaminants and thyroid hormones at 7 months of age.

**Conclusion:**

Overall, there is little evidence that the environmental contaminants analyzed in this study affect thyroid hormone status in Inuit mothers and their infants. The possibility that PCP may decrease thyroxine levels in neonates requires further investigation.

Tyroid hormones (TH) play several essential roles for brain maturation in humans. Adequate levels are necessary at specific times in certain regions of the brain for the normal development of the nervous system during fetal and neonatal periods (Porterfield and Hendrich 1993). Maternal TH are the only source of TH for the developing brain of the fetus during the first trimester of pregnancy, at the end of which the fetal hypothalamic–pituitary system initiates its own TH synthesis and secretion (Calvo et al. 2002). Studies have demonstrated that maternal TH can regulate gene expression in the fetal cortex, but their action on the developing brain remains unclear (Dowling and Zoeller 2000). Any alteration of maternal and/or fetal TH availability during critical periods of TH-dependent action may have a detrimental impact on the developing fetal brain, as previously demonstrated in animal and human studies (Morreale de Escobar et al. 2000; Pop et al. 2003).

Prenatal exposure to polychlorinated biphenyls (PCBs), a group of persistent and lipophilic organochlorine compounds (OCs), has been associated with neurodevelopmental deficits in childhood in most cohort studies focusing on developmental neurotoxicity of PCBs (Jacobson and Jacobson 1996; Stewart et al. 2008). Animals studies have reported detrimental effects of *in utero* exposure to PCBs on cognitive (Schantz and Widholm 2001) and motor functions (Schantz et al. 1997) as well as on the auditory system (Goldey et al. 1995). At the biochemical level, several PCB congeners disrupt the thyroid function, principally by lowering plasma thyroxine (T_4_) (Brouwer et al. 1995) and greatly depressing T_4_ concentrations in brain tissue in rats exposed prenatally (Morse et al. 1996).

Hexachlorobenzene (HCB) has been used as a fungicide and is also a by-product of the manufacturing of chlorinated solvents and metallurgical processes. HCB is found worldwide because of its long-range atmospheric transport, is very persistent, and tends to accumulate in adipose tissues. Exposure to HCB in rats can produce several effects including reduced T_4_ plasma levels and hypothyroidism (van Raaij et al. 1993). HCB has been shown to interfere with T_4_ uptake into the cerebrospinal fluid in rats (van Raaij et al. 1994).

Transport of T_4_ across the blood–brain barrier by transthyretin (TTR) is one mechanism by which TH reaches the developing fetal brain (Schreiber et al. 1995) and also facilitates maternal transfer across the placenta (Brouwer et al. 1998). Some chlorinated phenolic compounds (CPC) such as the hydroxylated metabolites of PCBs (HO-PCBs) and pentachlorophenol (PCP) possess a greater *in vitro* binding affinity for TTR than the natural ligand T_4_ (van den Berg 1990). Furthermore, HO-PCBs and PCP can strongly inhibit TH sulfation (Schuur et al. 1998), which along with desulfation appear to be very important pathways regulating circulating TH concentrations in the fetus (Darras et al. 1999).

Results from epidemiologic studies among pregnant women are scarce, but suggest potential effects of OCs on thyroid economy (Chevrier et al. 2008; Koopman-Esseboom et al. 1994; Takser et al. 2005). In contrast, assessments of the relation between environmental contaminants and the thyroid status of newborns have resulted in equivocal findings, possibly because of differences in the biological matrices for exposure determination (blood, plasma, placenta, or breast milk), the contaminants measured, and the congener grouping as well as the timing of postnatal TH dosage (Maervoet et al. 2007). With regard to CPCs, only two studies have investigated their effects in newborns and found opposite results (Otake et al. 2007; Sandau et al. 2002).

Because the potential effects of thyroid-disrupting contaminants on maternal and neonatal thyroid status are not yet clear, we undertook a study to determine the relationship of background exposure to OCs and CPCs with TH status in pregnant women and their infants within the first year of the infant’s life. This study addresses this research question among the Inuit population of Nunavik because these communities are among the most heavily exposed to these contaminants on earth, mainly through sea mammal consumption.

## Materials and Methods

### Population

Nunavik is the northern region of Québec where approximately 9,500 Inuit live in 14 communities on the coast of the Hudson and Ungava Bays. All pregnant Inuit women from the three largest communities of the Hudson Bay coast of Nunavik were invited to participate in a study that aimed to document the effects of prenatal exposure to environmental contaminants on infant development.

From November 1995 to March 2001, a research assistant contacted each potential participant after her first prenatal visit with a midwife or a nurse. All pregnant women gave informed consent for themselves and for their infants before participating in the study. The study protocol was approved by the Nunavik Nutrition and Health Committee and by ethics committees at Laval University (Québec City, Québec, Canada) and Wayne State University (Detroit, Michigan). One prenatal and two postnatal (at 1 and 6 months) interviews were conducted to obtain sociodemographic and personal information from the mothers. The participation rate for this study was 69%. Among the 248 participating mother–infant dyads, 17.8% were excluded due to the adoption of the newborn, fetal or postnatal mortality, and loss to follow-up. Biological data on TH and OC concentrations were available for 204 pregnant women. Because TH levels slowly return to non-pregnancy levels after delivery and to ensure that measured levels of TH were not related to postpartum thyroid dysfunction, which occurs between 2 and 12 months postpartum (Stagnaro-Green 2004), mothers from whom the blood sample was drawn 31 days after delivery were excluded from the statistical analysis (*n* = 71). Pregnant women who declared using medication for thyroid diseases were also excluded (*n* = 4). Biological data on TH and OC concentrations were available for 108 infants at birth and 175 infants at 7 months of age. Neonates born prematurely and/or with low birth weight were also excluded because transient neonatal hypothyroxinemia may be induced by prematurity (< 37 weeks gestation, *n* = 6) and low birth weight (< 2,500 g, *n* = 1) (Fisher 1998).

### Blood sampling and laboratory procedures

A 12.5-mL blood sample was collected from participating women at delivery or within the subsequent weeks (median = 2 days). A 30-mL blood sample was collected from the umbilical cord after it was severed, and a 5-mL blood sample was obtained at 7 months postpartum (median = 6.9 months). OC, CPC, and TH levels were measured in maternal, cord, and infant plasma. Selenium and plasma lipid concentrations were also measured in samples for confounding assessment.

Blood samples were collected in vials containing EDTA as the anticoagulant and centrifuged (10 min, 5,000 rpm), and the plasma transferred into glass vials prewashed with hexane. Plasma samples were frozen at −80°C until time of analysis. We measured concentrations of the 14 most frequently detected PCB congeners in human populations [IUPAC (International Union of Pure and Applied Chemistry) nos. 28, 52, 99, 101, 105, 118, 128, 138, 153, 156, 170, 180, 183, 187] and HCB in the purified extracts using an HP-5890 series II gas chromatograph (Hewlett-Packard, Palo Alto, CA, USA) equipped with dual-capillary columns (HP Ultra and Ultra II; Hewlett-Packard) and dual Ni-63 electron capture detectors (Hewlett-Packard). Detailed laboratory procedures and quality control procedures for OCs were described previously by Rhainds et al. (1999) and Muckle et al. (2001), respectively. Limits of detection for OCs were 0.02 μg/L.

We determined concentrations of CPCs by extracting the plasma samples on an Oasis HLB (540 mg; Waters Corp., Milford, MA, USA) solid phase extraction column according to a modified method by Sandau et al. (2003). Extraction and cleanup were completed on a Zymark Rapidtrace Automated SPE workstation (Zymark Corp., Hopkinton, MA, USA). CPCs were eluted with dichloromethane:methanol (9:1) in a third fraction on a prepacked Florisil column (1 g; Alltech, Lexington, KY, USA). After evaporation, the compounds were derivatized using diazomethane in hexane according to the method of Sandau (2000). The derivatized fraction was then cleaned up on an activated silica/acidic silica column before evaporation, addition of recovery standard, and quantification by gas chromatography/high resolution mass spectrometry. HO-PCB results are presented as a sum of the 11 major HO-PCBs (4-HO-CB-107, 3-HO-CB-153, 4-HO-CB-146, 3′-HO-CB-138, 4-HO-CB-163, 4-HO-CB-187, 4-HO-CB-202, 4′-HO-CB-172, 4-HO-CB-193, 4′-HO-CB-199, and 4-HO-CB-208). The limit of detection for those compounds varied between 2 and 6 ng/L.

We determined total cholesterol, free cholesterol, and triglycerides in plasma by standard enzymatic procedures, whereas we quantified phospholipids according to Takayama et al. (1977) using a commercial kit (Wako Pure Chemical Industries, Richmond, VA, USA). Concentrations of total plasma lipids were estimated according to the formula developed by Akins et al. (1989). Blood Se concentrations were determined using inductively coupled plasma mass spectrometry (PE Elan 6000; PerkinElmer Sciex, Concord, ON, Canada). The limit of detection for blood Se was 0.1 μmol/L.

We analyzed serum throid-stimulating hormone (TSH), free T_4_ (fT_4_), total triiodothyronine (T_3_), and thyroid-binding globulin (TBG), the major transport protein of TH, using radioimmunoassay methods. TSH, fT_4_, and T_3_ were measured on the Bayer Immuno 1 System (Bayer Corp., Tarrytown, NY, USA). TBG was determined on the Clinical Assays GammaDab system commercialized by DiaSorin (Stillwater, MN, USA).

### Statistical analysis

Contaminant concentrations and thyroid parameters were all log-transformed to satisfy criteria of normality. We used Pearson correlations to evaluate relations between contaminants, and simple and multiple linear regression models to evaluate the relationship between environmental contaminants, TH, and TBG in plasma. A value equal to half the limit of detection of the analytical method was attributed to nondetected contaminants in biological samples. Only PCB congeners with a detection frequency of at least 70% were considered. We evaluated exposure to PCB by several approaches. First, all PCB congeners were evaluated individually. Second, congeners were grouped according to their dioxin-like activity or their capacity to induce the microsomal enzymes UDP-GT (uridine diphosphate-glucuronosyl transferase), CYP1A (cytochrome P4501A), and CYP2B as proposed by Wolff et al. (1997). For the later grouping, the enzyme-inducing congeners included were PCBs 99, 118, 153, 180, and 187. Third, because all PCB congeners were highly correlated in the study population, PCB-153 was selected as a marker of exposure to all PCBs (Muckle et al. 2001). Indeed, in maternal plasma, intercorrelations among PCB congeners varied from 0.62 to 0.96. Similarly, in cord and infant plasma, intercorrelations among PCB congeners varied from 0.73 to 0.98 and 0.81 to 0.99, respectively. Correlations between PCB-153 and the sum of PCB inducers in maternal, cord, and infant plasma were all 0.99. Because results obtained using all PCB congeners or groupings did not change significantly from those obtained with PCB-153 (data not shown), only results for PCB-153 are presented.

We performed simple regressions between potential confounding variables and thyroid parameters to determine which covariate should be included in multiple linear regression analyses. Covariates associated at a *p*-value ≤ 0.10 were included in multiple regression models to assess their confounding influence. Covariates modifying the regression coefficient of the contaminants by > 10% with any of the TH were included in adjusted models for all TH parameters. The following covariates were evaluated in pregnant women: age at delivery, prepregnancy body weight, socioeconomic status [Hollingshead Index of Social Status (Hollingshead AB, unpublished data)], alcohol and cigarette consumption, fish consumption during pregnancy, and plasma Se concentration. Because age at delivery and parity were highly intercorrelated, only the former was retained in the multiple models, where appropriate, because it was a better predictor of TH concentrations in pregnant women. Nine women were not included in statistical analysis because of absence of information regarding alcohol consumption. Most variables evaluated as potential confounders in newborns were also tested in the models at 7 months of age. The primary caregiver’s score for socioeconomic status, maternal alcohol and cigarette consumption, maternal fish consumption during pregnancy, sex, breast-feeding status, as well as Se level in cord blood were tested for confounding at both ages. Gestational age and birth weight were also highly intercorrelated in newborns. erefore, only the former was included in multiple regression models, where appropriate, because it was a better predictor of TH concentrations. Six newborns were excluded due to unmeasured cord selenium concentrations, and 15 infants were excluded for lack of information regarding breast-feeding status. Because breast-feeding is an important source of postnatal exposure, an interaction term between OC concentrations and breast-feeding status at 7 months of age was also tested in multiple regression models. Statistical models assessing the relationship between the lipophilic contaminants PCB-153 or HCB and thyroid parameters were further adjusted for total plasma lipid concentrations because Schisterman et al. (2005) have shown that this approach is less prone to estimate bias compared with lipid standardization. Adjustment for lipid content was not done for CPCs because they are bound to plasma proteins (Sandau et al. 2000).

A bilateral *p*-value < 0.05 was considered statistically significant. Database management and variable transformation were performed with SPSS (version 2.0; SPSS Inc., Chicago, IL, USA) whereas statistical analyses were conducted with SAS (version 9.1; SAS Institute Inc., Cary, NC, USA).

## Results

Table 1 lists the characteristics of included and excluded pregnant Inuit women and their infants at birth and at 7 months of age. Sociodemographic characteristics and exposure to OCs (data not shown) were similar between the pregnant women who were included and and those excluded from the study. In accordance with the exclusion criteria, newborns included in the study had a longer gestational age and were heavier. Percentages of breast-feeding at birth and at 7 months of age were significantly greater in infants included than in those excluded. ere was no significant difference in exposure to OCs between included and excluded infants at birth and at 7 months of age (data not shown).

Table 2 presents descriptive statistics for OCs and CPCs in maternal, cord, and infant plasma. PCB-153 is the most prevalent congener in this population the mean concentration of PCB-153 was higher in pregnant women, followed by infant and cord plasma levels. The correlation on a lipid basis between maternal and cord plasma PCB-153 was very high (*r* = 0.94), but only moderate between maternal and infant concentrations (*r* = 0.41) and cord and infant concentrations (*r* = 0.46). Mean lipid-adjusted HCB concentrations were similar for newborn, infant, and maternal plasma samples. As for PCB-153, the correlation between maternal and cord plasma HCB was high (*r* = 0.86), but in the moderate range between maternal and infant concentrations (*r* = 0.33) and cord and infant concentrations (*r* = 0.36). The mean wet weight concentration of HO-PCBs was higher in pregnant women than in cord plasma, and levels were highly intercorrelated (*r* = 0.96). Most of the HO-PCBs detected in mothers and newborns were moderately correlated with PCB-153 (*r* = 0.21–0.78, median: newborn *r* = 0.63; maternal *r* = 0.53) on a wet weight basis. PCP was detected in all maternal and umbilical cord plasma samples. Levels were higher in umbilical cord than in maternal plasma and were also very highly correlated (*r* = 0.85). Intercorrelations of contaminants in maternal, umbilical cord, and infant plasma are also presented in Table 2. In maternal and cord samples, PCB-153 concentrations were highly correlated with HO-PCBs and moderately with HCB. Also, PCP was weakly correlated with the other contaminants. Concentrations of PCB-153 and HCB were highly correlated in infant plasma.

A large proportion of the mothers had TSH values outside the TSH euthyroid reference range (0.4–2.5 mIU/L) (Demers and Spencer 2002). Sixteen percent (*n* = 20) had TSH concentrations below the lower limit whereas 10% (*n* =13) had values higher than the reference range. ere were no significant differences in OC concentrations between mothers outside and within the TSH euthyroid refence range. Most infants at birth and at 7 months of age had TSH and fT_4_ levels within the normal range (Demers and Spencer 2002; Hume et al. 2004).

The associations between OCs and CPCs and the thyroid parameters documented in pregnant women at delivery are presented in Table 3. Simple regression models showed a significant positive association between HO-PCBs and T_3_. Simple and multiple regressions between contaminants and TSH as well as fT_4_ were not significant. Adjustment for confounding factors strengthens the positive association between HO-PCBs and T_3._

Table 4 shows simple and multiple linear associations between thyroid parameters measured in umbilical cord blood and OCs or CPCs levels in cord and maternal plasma. In newborns at birth, no significant association was observed between cord blood contaminants and TH concentrations in univariate models. However, negative associations were found for umbilical cord and maternal PCB-153 with TBG concentrations after control for confounders. A negative association between cord fT_4_ and maternal PCP, but not cord PCP, was found before and after controlling for confounders. In the subsample analysis of cord CPC, characteristics of newborns included were similar, except that those included had a higher birth weight (included: mean = 3,758 g, excluded: mean = 3,508 g, *p* = 0.006). Also, in the subsample analysis of maternal CPC, characteristics of mothers and newborns were similar, except that maternal and cord Se concentrations were lower (mothers included: mean = 1.09 μmol/L, excluded: mean = 1.44 μmol/L, *p* = 0.01; newborns included: mean = 3.11 μmol/L, excluded: mean = 3.84 μmol/L, *p* = 0.04). Concentrations of T_3_ in cord blood were not associated with any of the contaminants under study. A significant negative association between PCB-153 and TSH was obtained in simple linear regression for infants 7 months of age (Table 5). However, the association was no longer significant after adjustment for breast-feeding status. Interaction terms between OCs and breast-feeding status were not significant. The associations were not significant after stratification for breast-feeding status (data not shown).

## Discussion

In the present study, we investigated the potential negative effects of OCs and CPCs on the circulating concentrations of TH among Inuit women and their infants. Overall, the results show that plasma concentration of OCs and CPCs were not significant predictors of TSH and TH concentrations in this population of pregnant women and their offspring. However, maternal levels of PCP were negatively associated with fT_4_ concen- trations in umbilical cord blood of neonates, suggesting that this compound may reduce the transfer of maternal T_4_ across the placenta to the developing fetus.

As reported in most studies [reviewed by Maervoet et al. (2007)], an association between PCB-153 and TH concentrations was not found in Inuit neonates at birth. Similarly, no association was seen in these infants at the 7-months follow-up. Also, these data did not corroborate the relationship between PCB congeners and TSH or TH levels seen in the CHAMACOS (Center for the Health Assessment of Mothers and Children of Salinas) study (Chevrier et al. 2007), in any of the exposure matrices (maternal, cord, or infant plasma). ese associations were observed even though the maternal PCB-153 concentrations were almost 20 times lower than those in the present study. Difference in the profile of other PCB congeners in that population may provide one explanation for the discrepancy in results between the CHAMACOS study and the present study. Another possible important difference between both populations is the iodine intake. Indeed, iodine, which is essential for thyroid function, is found in high concentration in some seafood products. erefore, exposure to iodine and OCs co-occurs in individuals such as the Nunavik Inuit, who consume a large amount of marine products. Therefore, not taking into account urinary iodine concentrations in epidemiologic studies aiming to investigate this research question may partly explain the inconsistent results.

The absence of an association between HCB and umbilical cord fT_4_ concentrations in this study are not consistent with a previous finding of significant positive associations in another sample of Inuit neonates and in newborns from the Lower North Shore of the St. Lawrence River (Dallaire et al. 2008). The lower statistical power of the present study as well as a difference in adjustment of confounders may account for the differential results. However, the absence of relationship between HCB and fT_4_ found in the present study are in agreement with two other epidemiologic studies among neonates (Ribas-Fito et al. 2003; Takser et al. 2005).

The absence of an association between PCB-153 and thyroid parameters in pregnant Inuit women was similar to that reported in the second Faroe Islands cohort (Steuerwald et al. 2000). However, two other epidemiologic studies among pregnant women found a significant negative association of PCBs with T_3_ and/or tT_4_ status (Koopman-Esseboom et al. 1994; Takser et al. 2005) whereas, among Mexican-American pregnant women, three PCB congeners (44, 52, and 183) were negatively related to fT_4_ concentrations (Chevrier et al. 2008). It is possible that the effects observed in these three studies were mostly attributable to the exposure to polychlorinated dibenzo-*p*-dioxins and polychlorinated dibenzofurans, which are usually higher in urban settings than in isolated populations mostly exposed to environmental contaminants through fish and marine mammal consumption. As previously mentioned, high dietary iodine intake in fish-eating populations may also explain the lack of association between PCB-153 and thyroid hormone status in mothers (Dallaire et al. 2008).

HCB body burden was not associated with thyroid hormone profile in Inuit pregnant women at delivery. Two other studies investigated whether exposure to low background concentrations of HCB was related to TH levels during pregnancy. An inverse relationship between plasma HCB concentrations and T_3_ levels was reported among Canadians (Takser et al. 2005), and negative associations between HCB concentrations and fT_4_ and tT_4_ levels were found in the CHAMACOS study (Chevrier et al. 2008). However, the authors were not able to determine whether effects on TH concentrations were related to PCBs or HCB plasma concentrations because these exposures were highly correlated.

In the present study we also documented concentrations of HO-PCBs and PCP because several *in vitro* studies have revealed their potency in competitively inhibiting T_4_ binding to TTR (van den Berg 1990). This T_4_-binding protein is implicated in the delivery of maternal T_4_ to the fetus across the placenta (McKinnon et al. 2005) as well as the transport of T_4_ from blood into cerebrospinal fluid during brain maturation (Southwell et al. 1993). However, experiments with TTR-null mice have shown that TTR is not essential for T_4_ distribution in brain (Palha 2002). Nevertheless, as would be predicted from toxicologic data, a negative association was found between maternal PCP plasma concentrations and umbilical cord fT_4_ levels, but not with umbilical cord PCP, which has two times the binding affinity of the natural ligand T_4_ for TTR (van den Berg 1990). Moreover, it was the predominant CPC in the Inuit population, and concentrations were mostly higher in umbilical cord plasma than in maternal plasma. Taken together, these findings seem to indicate that PCP may impede the transfer of maternal T_4_ to the fetus across the placental barrier to a certain extent by inhibiting maternal T_4_ binding to TTR. is mechanism may also have favored the transport of maternal PCP to the fetal circulation, which may explain the higher concentration of this compound in cord blood compared with maternal blood. The relation between maternal PCP and neonate fT_4_ concentrations was no longer evident at the later age, because there was no association between maternal or cord PCP and fT_4_ levels when the infants were 7 months of age (data not shown). The rapid maturation of the hypothalamus–pituitary–thyroid axis after the severing of the umbilical cord, which leads to the complete independence of the newborn from a maternal source of fT_4_, may explain such findings. However, these results should be interpreted with caution because no association was found with cord blood PCP concentrations even though they were highly correlated with maternal PCP levels (*r* = 0.85). Also, plasma Se concentrations of mothers and newborns included in the subsample analysis were lower than in the sample used for OCs (*n* = 80). Two other epidemiologic studies have looked at the association between CPC and TH concentrations in neonates. In another sample of Inuit newborns, Sandau et al. (2002) found a negative correlation between the sum of PCP and 14 HO-PCBs with cord fT_4_, whereas Otake et al. (2007) reported a positive correlation between 6 HO-PCBs and neonatal blood-spot fT_4_ obtained on a filter paper. Other studies are needed to ascertain the potential effects of CPCs on neonatal TH concentrations, because the small sample size in the present study limits its conclusions.

The increase of T_3_ with concentrations of HO-PCBs in pregnant women may be attributable to selective or combined inhibition of sulfotransferase and type I deiodinase (D_1_) activities, as these metabolites have been shown to competitively inhibit D_1_ and diiodothyronine sulfotransferases actions (Schuur et al. 1998). Indeed, in adults, sulfotransferases irreversibly inactivate TH actions and facilitate D_1_ deiodination for complete degradation in the liver (Visser 1996). Interestingly, in fetuses sulfated T_4_ and T_3_ are elevated compared with adult concentrations and have been proposed to serve as a reservoir of inactive TH for subsequent activation by sulfotransferases at specific developmental stages (Darras et al. 1999). is difference in TH metabolism between fetuses and adults, especially for sulfotransferase and deiodinase activities, may explain the absence of HO-PCB effects on cord T_3_ compared with maternal T_3_.

These findings suggest that the synthesis or metabolism of transport proteins may be affected by environmental contaminants. Indeed, the negative association between PCB-153 and serum TBG concentration could by mediated by a diminution of TBG synthesis by the liver. Interestingly, we already reported a similar association in a population of neonates from the Lower North Shore of the St. Lawrence River (Dallaire et al. 2008). Because commercial fT_4_ immunoassays overestimate the fT_4_ levels at high TBG concentration and underestimate it at low TBG concentrations (Demers and Spencer 2002), these results may indicate that contaminants affecting TBG concentrations introduce bias in the assessment of the effects of thyroid toxicants on fT_4_ levels. Most toxicologic studies have focused on the competitive binding assessment of environmental contaminants with T_4_, without evaluating their ability to modify transport protein synthesis. Consequently, the results cannot be corroborated on biological mechanisms because toxicologic data are unavailable. Nevertheless, such an effect remains plausible. By analogy, Richardson et al. (2008) have recently shown that polybrominated diphenyl ether congener 47 has the capacity to decrease TTR mRNA expression in the liver by an unidentified mechanism. An assessment of transport protein biosynthesis modification by environmental contaminants is needed to clarify this question.

## Conclusion

In summary, in this study we did not find similar effects of potential thyroid toxicants on TH homeostasis in mother and infant. Differential TH and contaminant metabolizing capacity between fetuses and adults may account for this discrepancy. There were also no associations between concentrations of PCB-153 and TH levels in Nunavik Inuit mothers and infants. However, a significant inverse relationship was found between maternal PCP levels and fT_4_ umbilical cord concentrations. This finding is in agreement with the hypothesis regarding the capacity of CPCs to competitively inhibit T_4_ binding to TTR in humans. However, this result must be interpreted with caution because it was found in a subsample of the neonate population and consequently needs to be corroborated in larger studies. Nevertheless, epidemiologic studies of pregnant women suffering from hypothyroxinemia (low fT_4_) have shown that reduced transfer of maternal fT_4_ during fetal brain maturation can negatively affect the neurocognitive function in infants (Pop et al. 1999, 2003). Results from the neurodevelopment assessment of the participating infants in this cohort will allow us to determine whether a reduction of fT_4_ concentrations during fetal development, perhaps mediated by PCP, may induce deficits similar to those observed in the offspring of hypothyroxinemic pregnant women.

## Figures and Tables

**Table 1:** Characteristics of included and excluded mothers and their infants.

	Included		Excluded		
Characteristic	No.	Mean ± SD	No.	Mean ± SD	*p*-Value
Maternal					

Age (years)	120	24.9 ± 5.9	119	25.2 ± 5.7	0.73
Marital status (% single)	120	31.0	128	27.0	0.38
Education (years)	120	8.9 ± 1.7	128	9.0 ± 1.8	0.68
Prepregnancy weight (kg/m^2^ )	120	61.1 ± 11.9	123	61.9 ± 12.0	0.62
Parity	120	2.0 ± 1.9	128	2.1 ± 1.8	0.91
No. of cigarettes/day (%)					
0	12	10	12	9	0.87
1–10	59	49	62	48	0.91
11–24	38	32	46	36	0.48
≥ 25	11	9	11	6	0.39
Average absolute alcohol/drinking day (oz)^a^	120	0.76 ± 0.8	95	0.70 ± 0.66	0.53
Plasma selenium (μmol/L)	86	1.34 ± 0.35	83	1.40 ± 0.38	0.55
No. of meals of fish/week	120	3.6 ± 4.3	95	3.1 ± 3.5	0.30

Neonate					

Gestational age (weeks)	95	39.1 ± 1.3	135	38.2 ± 2.0	< 0.01
Sex (% male)	95	62.1		53.3	0.18
Weight (g)	95	3,616 ± 449	143	3,301 ± 624	< 0.01
Height (cm)	94	50.9 ± 1.7	116	49.7 ± 2.5	< 0.01
Breast-fed at birth (%)	77	54	171	25	< 0.01
Umbilical cord Se (μmol/L)	95	3.68 ± 1.56	12	3.42 ± 0.79	0.56

Infant					

Age (days)	130	235 ± 70	61	228 ± 51	0.47
Sex (% male)	130	60.8	100	52.0	0.18
Weight (kg)	123	9.7 ± 1.3	47	9.6 ± 1.3	0.77
Height (cm)	125	67.4 ± 3.5	51	67.7 ± 3.1	0.60
Breast-fed at 7 months (%)	130	51	118	16	< 0.01

a 0.5 oz absolute alcohol corresponds to 12 oz beer, 6 oz wine, or 1.5 oz liquor.

**Table 2:** Concentrations of organochlorines and chlorinated phenolic compounds in plasma from pregnant Inuit women and their infants at birth and at 7 months of age.

			Lipid standardized concentrations (μg/kg)			Wet weight concentrations (μg/L)^a^			Pearson correlations		
Analytes	No.	Percent detected	Geometric mean	95% CI	Range	Geometric mean	95% CI	Range	HCB	∑HO-PCBs	PCP
Maternal											

PCB-153	120	100	107.7	93.2–124.5	15.6–709.0	0.89	0.77–1.04	0.01–6.12	0.69#	0.84#	0.34
HCB	120	100	41.1	36.5–46.1	6.7–352.6	0.34	0.30–0.39	0.05–2.78		0.65#	0.32
∑HO-PCBs^a^	25	—		—	—	316	245–409	109–1,517			0.36
PCP^a^	25	100		—	—	931	772–1,123	241–2,898			

Cord											

PCB-153	95	100	83.1	70.4–98.0	12.0–550.9	0.20	0.17–0.24	0.03–2.42	0.70#	0.82#	0.17
HCB	95	100	42.9	38.0–48.4	10.6–184.5	0.11	0.09–0.12	0.02–0.83		0.60#	0.12
∑HO-PCBs^a^	41	—		—	—	246	195–309	65–1,358			0.23
PCP^a^	41	100		—	—	1,078	923–1,267	300–2,913			

Infant											

PCB-153	130	98.0	90.5	72.9–112.3	< LOD–1387.3	0.45	0.36–0.57	< LOD–8.54	0.92#	—	—
HCB	130	95.9	41.5	34.1–50.5	< LOD–446.7	0.21	0.17–0.26	< LOD–2.75			—

Abbreviations: CI, confidence interval; LOD, Limit of detection.

a ∑HO-PCBs and PCP concentrations are expressed in pg/g wet weight.

# *p* < 0.001.

**Table 3:** Linear regression models of thyroid hormones and TBG plasma levels with organochlorines and chlorinated phenolic compounds in plasma from pregnant Inuit women.

Analyte	Thyroid parameter	No.	Univariate β	Standardized β
PCB-153 (μg/L)^a^	TSH (mIU/L)	120	−0.05	0.05
	fT_4_ (pmol/L)	120	−0.01	−0.05
	T_3_ (nmol/L)	120	0.01	0.08
	TBG (nmol/L)	120	0.03	0.13

HCB (μg/L)^a^	TSH (mIU/L)	120	0.03	0.13
	fT_4_ (pmol/L)	120	0.02	0.07
	T_3_ (nmol/L)	120	0.02	0.07
	TBG (nmol/L)	120	0.05	0.15

∑HO-PCBs (pg/g wet weight)	TSH (mIU/L)	25	−0.14	0.08
	fT_4_ (pmol/L)	25	−0.05	−0.40
	T_3_ (nmol/L)	25	0.16**	0.57*
	TBG (nmol/L)	25	0.14	0.30

PCP (pg/g wet weight)	TSH (mIU/L)	25	0.35	0.27
	fT_4_ (pmol/L)	25	−0.11	−0.22
	T_3_ (nmol/L)	25	0.10	0.16
	TBG (nmol/L)	25	0.08	0.06

Standardized β adjusted for age at delivery, alcohol (average absolute alcohol/drinking day), and cigarette (cigarettes/day) consumption during pregnancy.

a Also adjusted for serum lipid concentrations.

* *p* < 0.05.

** *p* = 0.01.

**Table 4:** Linear regression models of thyroid hormone and TBG levels in umbilical cord with organo chlorine and chlorinated phenolic compounds in maternal and umbilical cord plasma.

Analyte	Thyroid parameter	Cord contaminants			Maternal contaminants		
		No.	Univariate β	Standardized β	No.	Univariate β	Standardized β
PCB-153 (μg/L)^a^	TSH (mIU/L)	95	0.01	−0.05	80	0.06	0.02
	fT_4_ (pmol/L)	95	−0.02	−0.14	80	−0.02	−0.14
	T_3_ (nmol/L)	95	−0.01	−0.04	80	0.03	0.04
	TBG (nmol/L)	95	−0.05	−0.26**	80	−0.05	−0.25**

HCB (μg/L)^a^	TSH (mIU/L)	95	−0.02	−0.09	80	−0.02	−0.10
	fT_4_ (pmol/L)	95	0.00	−0.04	80	0.00	−0.02
	T_3_ (nmol/L)	95	−0.06	−0.18	80	0.00	−0.09
	TBG (nmol/L)	95	−0.03	−0.15	80	−0.01	−0.15

∑HO-PCBs (pg/g wet weight)	TSH (mIU/L)	41	−0.04	−0.12	19	0.06	0.05
	fT_4_ (pmol/L)	41	0.00	−0.08	19	0.02	0.05
	T_3_ (nmol/L)	41	0.05	0.10	19	0.15	0.17
	TBG (nmol/L)	41	−0.06	−0.25	19	0.00	−0.02

PCP (pg/g wet weight)	TSH (mIU/L)	41	−0.01	0.02	19	−0.01	−0.05
	fT_4_ (pmol/L)	41	−0.05	−0.20	19	−0.13**	−0.59*
	T_3_ (nmol/L)	41	−0.18	−0.31	19	−0.07	−0.11
	TBG (nmol/L)	41	−0.09	−0.28	19	−0.11	−0.34

Standardized β adjusted for gestational age, cord selenium, and maternal cigarette (cigarettes/day) consumption during pregnancy.

a Also adjusted for serum lipid concentrations.

* *p* < 0.05.

** *p* = 0.01.

**Table 5:** Linear regression models of thyroid hormone and TBG plasma levels with organochlorines in plasma from 7-month-old Inuit infants.

Analyte	Thyroid parameter	No.	Univariate β	Standardized β
PCB-153 (μg/L)	TSH (mIU/L)	130	−0.09*	−0.23
	fT_4_ (pmol/L)	130	0.02	0.11
	T_3_ (nmol/L)	130	−0.01	−0.12
	TBG (nmol/L)	132	0.02	−0.03
HCB (μg/L)	TSH (mIU/L)	128	−0.06	−0.13
	fT_4_ (pmol/L)	128	0.02	0.09
	T_3_ (nmol/L)	128	−0.01	−0.11
	TBG (nmol/L)	130	0.03	0.03

Standardized β adjusted for breast-feeding status at 7 months and serum lipid concentrations.

* *p* < 0.05.
